# Psychiatric Disorders in Refugees and Internally Displaced Persons After Forced Displacement: A Systematic Review

**DOI:** 10.3389/fpsyt.2018.00433

**Published:** 2018-09-21

**Authors:** Naser Morina, Aemal Akhtar, Jürgen Barth, Ulrich Schnyder

**Affiliations:** ^1^Department of Consultation Psychiatry and Psychosomatic Medicine, University Hospital Zurich, University of Zurich, Zurich, Switzerland; ^2^School of Psychology, University of New South Wales, Sydney, NSW, Australia; ^3^Institute for Complementary and Integrative Medicine, University Hospital Zurich, University of Zurich, Zurich, Switzerland

**Keywords:** mental health, psychiatric disorders, refugees, internally displaced people, epidemiology, war, systematic review

## Abstract

**Background:** Protracted armed conflicts not only shape political, legal, and socio-economic structures, but also have a lasting impact on people's human migration. In 2017, the United Nations High Commissioner for Refugees reported an unprecedented number of 65.6 million individuals who were displaced worldwide as a result of armed conflicts. To date, however, little is known about these people's mental health status. Therefore, we conducted a systematic review of the prevalence of psychiatric disorders among forcibly displaced populations in settings of armed conflicts.

**Methods:** We undertook a database search using Medline, PsycINFO, PILOTS, and the Cochrane Library, using the following keywords and their appropriate synonyms to identify relevant articles for possible inclusion: “mental health,” “refugees,” “internally displaced people,” “survey,” and “war.” This search was limited to original articles, systematic reviews, and meta-analyses published after 1980. We reviewed studies with prevalence rates of common psychiatric disorders—mood and anxiety disorders, psychotic disorders, personality disorders, substance abuse, and suicidality—among adult internally displaced persons (IDPs) and refugees afflicted by armed conflicts.

**Results:** The search initially yielded 915 articles. Of these references 38 studies were eligible and provided data for a total of 39,518 adult IDPs and refugees from 21 countries. The highest prevalence were for reported for post-traumatic stress disorder (3–88%), depression (5–80%), and anxiety disorders (1–81%) with large variation. Only 12 original articles reported about other mental disorders.

**Conclusions:** These results show a substantial lack of data concerning the wider extent of psychiatric disability among people living in protracted displacement situations. Ambitious assessment programs are needed to support the implementation of sustainable global mental health policies in war-torn countries. Finally, there is an urgent need for large-scale interventions that address psychiatric disorders in refugees and internally displaced persons after displacement.

## Introduction

“War should be understood as an *actual, intentional*, and *widespread* armed conflict between political communities […], defined as those entities which either are states or intend to become states […]” (1). Whether considered from a philosophical, sociological, or legal perspective, war remains one of the most complex and devastating human enterprises (1). In 2016, according to the *Department of Peace and Conflict Research* at the University of Uppsala, 51 ongoing armed conflicts were reported worldwide and well over 100,000 people were killed in organized violence (2). Beyond this sole number, the entire ecology of war has dramatically changed over the past two decades. Altogether less frequent, armed conflicts have reached low- and middle-income countries (LMICs) more frequently and become predominantly intrastate and disproportionately protracted in nature (3).

Intrastate, irregular, and protracted armed conflicts have drastically influenced recent figures of global displacement (4). In its *2017 Global Trends Report*, the United Nations High Commissioner for Refugees (UNHCR) announced an estimated 10.3 million newly displaced individuals—Syria being the most affected country—and an overall number of 65.6 million forcibly displaced people worldwide (5). While UNHCR refugee statistics have demonstrated a substantial stabilization of the number of out-of-country refugees over the past 10 years, numbers of internally displaced people (IDP) have reached unparalleled levels representing more than 65% of the displaced population globally (6). In 2016 alone, conflict and violence gave rise to 6.9 million new IDPs, which disproportionately came from LMICs.

Organized violence has profound and catastrophic structural effects on already fragile developing countries, where 84% of the world's refugees live (5); political and economic structures are undermined, laws are overstepped, fundamental rights of individuals are often abused, and healthcare services shattered (3, 7, 8). Quantifying the magnitude of these consequences on people undoubtedly remains an intricate challenge (7). However, numerous public health studies in complex humanitarian settings have shown that armed conflicts critically affect mental health (6, 9–11).

Reliable assessments of mental health needs in humanitarian settings should be viewed as a public health priority. In fact, no mental health policies can be efficiently implemented without an accurate assessment. This seems particularly true today: numbers of global displacement related to conflicts are increasing dramatically, common camp-based models of displaced populations are becoming outdated—60% of IDPs are currently living in urban areas without any international protection—and the heavy burden of psychiatric diseases in LMICs are being acknowledged (3, 12).

Based on the assumption that conflict-affected populations will be traumatized, most studies have singularly focused on PTSD whilst largely ignoring other psychiatric disorders. Considering psychological trauma or depression as main research objectives in settings of generalized violence or unresolved conflicts is a major intellectual misapprehension. It fails to appreciate the extent of *global* repercussions on mental health, that is, the risks of intense psychological distress in otherwise healthy individuals and increased neuropsychiatric diseases or the worsening of chronic mental illnesses due to an aggravated lack of medical resources (13–15). Additionally, these risks interact with several environmental factors that directly influence mental health such as forced evacuation of homes and separation from family members, interpersonal tensions, or loss of employment opportunities (13–15).

Two previous systematic reviews have evaluated prevalence rates of psychiatric disorders in refugee populations. Steel et al. (16) conducted a systematic review and meta-analysis finding higher prevalence rates of PTSD and depression in conflict-displaced refugees globally. Another review from Ezard et al. (17) observed the rates of substance abuse among refugees displaced by conflict and reported on associated risk factors and outcomes. There is still a paucity of data on the epidemiology of mental illness in populations displaced by armed conflicts. This is especially true for less common psychiatric disorders such as psychotic disorders. Therefore, we conducted a systematic review to summarize the prevalence of common psychiatric disorders, as well as more severe—uncommon—psychiatric disorders in IDPs and refugees still residing in LMIC's. We also report on the methods of assessing mental illness, number, and types of traumatic events, and the duration of the displacement experience.

## Methods

To ensure the highest standardized methods of reviewing process, the conduct of this research was guided by the PRISMA guidelines for systematic reviews and meta-analyses (18).

### Search strategy

**Figure 1:**
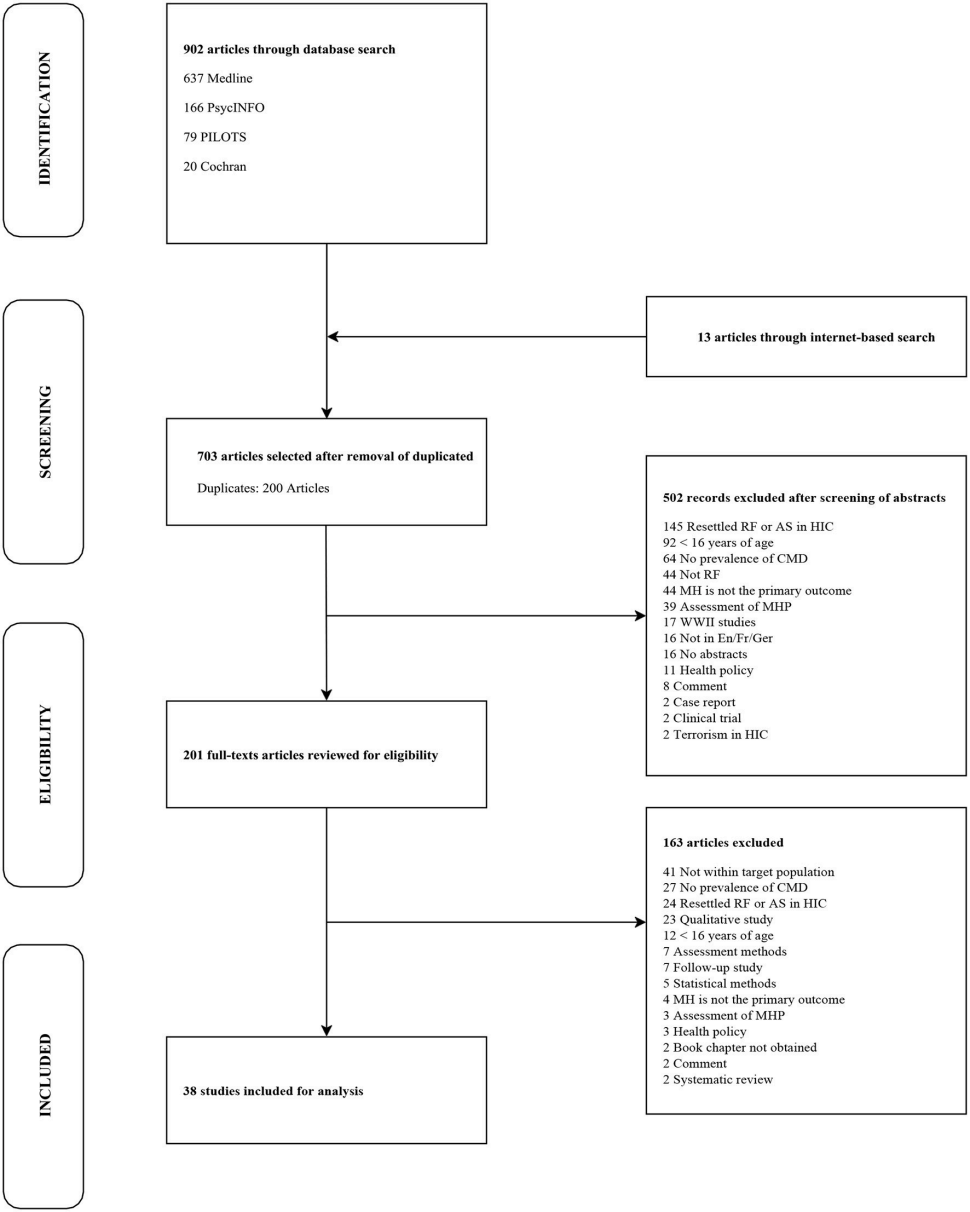
Research strategy to identify eligible articles examining common mental disorders among IDPs and people in protracted refugee situations. AS, Asylum Seekers; CMD, Common Mental Disorders; En/Fr/Ger, English/French/German; HIC, High Income Countries; MH, Mental Health; MHP, Mental Health Programs; RF, Refugees; WWII, World War II.

We undertook a sequential database search using MEDLINE via PubMed, PsycINFO, PILOTS, and the Cochrane library of systematic reviews. Medical Subject Headings with related text-based search terms were used with a combination of the following terms and concepts: “*mental health*,” “*refugees*,” “*prevalence*,” and “*war.”* In addition, articles indexed by “*internally displaced people”* were identified using the method of single keywords. Similarly, in the PILOTS database, an online index collecting the literature on PTSD and mental health consequences of traumatic events (19), a combination of single keywords and their synonyms was used to identify pertinent studies: “*mental health,”* “*refugees,”* “*internally displaced people,”* “*war,”* “*prevalence,”* and “*humanitarian settings.”* Relevant gray literature (unpublished articles, international reports, or non-governmental epidemiological surveys) was retrieved through an internet search. Lastly, citations from relevant articles and systematic reviews were also screened. This initial process yielded an overall number of 915 articles: 902 were generated through systematized database search, while 13 were retrieved from the gray literature and article/systematic review bibliographies (Figure 1).

### Inclusion and exclusion criteria

Studies were considered eligible for the review if they assessed the prevalence of common mental disorders among internally displaced people and refugees afflicted by armed conflicts in a war region or unstable country. Original articles written in English, French, and German were included. The issue of internally displaced or refugee children and adolescents should be addressed separately (20–23) and therefore, an age limit of ≥16 was selected as an inclusion criterion. We also limited our research to articles published after 1980 as interests about global mental health in war-torn countries emerged after 1980 when mental health progressively became an integral part of the field of public health (24). The status of conflict and displacement were other important inclusion criteria. We only included studies that reported results from or were completed in countries where conflicts were unresolved at the time of investigation and if study samples consisted of populations in protracted displacement situations. However, the UNHCR definition of “*protracted refugee situations”*— ≥ 25,000 people gathered outside of their country of residence for ≥5 consecutive years—was not considered as part of the selection process (25). In this context, we did not restrict article selection according to the numbers of IDPs or refugees and years of displacement, since many authors did not report this information. Furthermore, we only included studies from LMICs defined according to the Gross National Income per capita classification proposed by the World Bank (26). We did so for two principal reasons. On the one hand, mental disorders among resettled refugees in Western high-income countries (HIC) have been investigated to a great extent in the light of PTSD, depression, and anxiety disorders (27). On the other hand, contextual factors such as a lower likelihood of being exposed to an ongoing conflict and its myriad consequences, resettlement in stable accommodations, greater economic or educational opportunities uniquely distinguish resettled refugees in HIC from displaced populations in LMICs (28). Finally, case reports, qualitative studies, systematic reviews, meta-analyses, and articles reporting consequences of natural disasters on mental health were excluded.

In order to precisely define the term *refugee*, the *1951 Refugee Convention* was considered (29, 30): a refugee is a person who has crossed the international borders of his or her country of origin due to a “well-founded fear of being persecuted for reasons of race, religion, nationality, membership of a particular social group or political opinion,” and “is unable to, or owing to such fear, is unwilling to avail himself of the protection of that country.” Related to this, *internally displaced people* are “persons or groups of persons who have been forced or obliged to flee […] their homes […], in particular as a result or in order to avoid the effects of armed conflict, situations of generalized violence, violations of human rights […] or human-made disasters, and who have not crossed an internationally recognized state border” (12).

### Study selection and data extraction

Study selection and data extraction were completed independently by two reviewers (LL & AA) in duplicate; disagreements between them were resolved by a third reviewer (NM). The following study characteristics were extracted: type and year of study, regionality, sample size, sampling method (when available), and type of assessment method of psychiatric disorders. With regard to assessment, different potential situations were examined: clinical diagnosis provided by a mental health care specialist according to the *Diagnostic and Statistical Manual of Mental Disorders* [DSM; (31)] or the *International Classification of Disease* [ICD; (32)], clinical assessment provided by a trained interviewer using various methods such as the *Harvard Trauma Questionnaire* [HTQ; (33)], or the *Hopkins Symptom Checklist-25* [HSCL-25; (34)], and mental health evaluation by means of a self-administered questionnaire. We extracted prevalence rates of mental disorders but scores of scales were not used. More importantly, we did not restrict the extraction to disorders such as PTSD, depression, or anxiety disorders but included also general neuropsychiatric illnesses and, when applicable, general distress. Additional data extracted covered: type, year, and country of conflict (the total duration of conflict was inferred); status, country, and length of displacement; average number of experienced and/or witnessed traumatic events; mean age of study participants, and male to female ratio. In order to compare countries in terms of severity of conflicts, levels of generalized violence, and political instability, we introduced the *Political Terror Scale* [PTS; (35)], an 5-level score developed by the American non-governmental organization (NGO) *Freedom House* in the early 1980s. This score uses data provided by *Amnesty International* and the *U.S. Department of State*. Countries with a PTS score equal to 1 are legally and politically stable, whereas countries with PTS scores equal to or greater than 4 are notably characterized by extensive human rights violations and increasing levels of politically and ideologically motivated violence.

## Results

We investigated 38 eligible original studies (Table 1) reporting data from a total population of 39,158 adult IDPs and refugees.

**Table 1:** Detailed analysis of studies meeting inclusion criteria (*N* = 38).

**Author**	**Design**	**Population of interest**	**Study characteristics**	**Outcome**	**Assessment methods**	**Results**	**PTS**
Mollica et al. (36)	Household survey	993 Cambodian refugees on the Thailand-Cambodian border (1975–1979)	Women: 61% Age: 18–34 y (57% of participants) Duration of displacement: >5 y (82% of participants)	PTSD Depression	HTQ HSCL-25 Administered by trained interviewers in native language	PTSD: 15% Depression: 55%	Cambodia (1979): 5/5
Allden et al. (37)	Semi-structured Interviews	104 Burmese political dissidents in Thailand registered with the UN High Commission (1999)	Women: 21% Age: >23 y (62% of participants)	PTSD Depression	HTQ HSCL-25 Administered by Burmese research assistants in native language	PTSD: 23.1% Depression: 38.5%	Burma (1999): 4/5
Mollica et al. (38)	Cross-sectional survey	534 Bosnian refugees in a camp established by the Croatian government near the city of VaraŽdin, Croatia (1996)	Women: 59% Mean age: 50 y Exposure to traumatic events: 6.5 Duration of displacement: >3 y (30% of participants)	PTSD Depression	HTQ HSCL-25 Administered by trained interviewers in native language	PTSD alone: 26% Depression alone: 39% Depression and PTSD: 21%	Croatia (1996): 3/5
Peltzer et al. (39)	Cross-sectional survey	100 Sudanese residing in 2 refugee camps (1), 44 ex-soldiers (2), 60 refugees attending camp health facilities (3), and 63 refugees attending traditional healers' (4) in Northern Uganda (1994-1996)	(1)Women: 58% Mean Age: 37 y (2)Mean Age: 32.3 Median exposure to traumatic events: 12 (3)Mean age: 30 y Mean exposure to traumatic events: 5 (4) Mean age: 40 y Median exposure to traumatic events: 7	PTSD Depression	HTQ HSCL-25 Administered by trained interviewers in native language	(1)PTSD: 32% Depression: N/A (2)N/A (3)PTSD: 13% Depression: 20% (4)PTSD: 26% Depression: 39%	Sudan (1996): PTS-A 4/5; PTS-S 5/5
Kozarić-Kovacić et al. (40)	Structured interviews	368 displaced individuals in several refugee camps near the city of Zagreb, Croatia (2000).	Women: 57% Mean age of men/women: 39 y/38 y Exposure to traumatic events: 3.8 Mean duration of displacement: 2.5 y	PTSD Alcohol dependence	Structured clinical interviews/Watson's PTSD (DSM-IIIR)/Modified HTQ based on previous work on field Structured clinical interviews / CAGE questionnaire Administered by general practitioners Clinical interviews and diagnoses made by a psychiatrist (DSM-IIIR)	PTSD: 50% (Men); 36 % (Women) Alcohol dependence: 60% (Men); 8% (Women) PTSD comorbid with Alcohol dependence: 70% (Men); 12% (Women)	Croatia (2000): 1/5
Lee et al. (41)	Structured questionnaires	170 North Korean refugees at the Yanbian Korean Autonomous Prefecture in China (1999)	Women: 52% Mean age: 32 y Exposure to traumatic events: 12 Duration of displacement: >1 y (76% of participants)	PTSD Depression Anxeity	HTQ HSCL-25 Administered by trained interviewers in native language	PTSD: 56% Depression: 81% Anxiety: 90%	North Korea (1999): 5/5
Tang et al. (42)	Refugee camp survey	80 Senegalese refugees from the Casamance region in The Gambia (2000)	Women: 49% Exposure to traumatic events: 11.28 Mean duration of displacement: 34 months	PTSD Depression Anxiety	HTQ HSCL-25 Administered by trained interviewers in native language	PTSD: 10% Depression: 59% Anxiety: 46%	The Gambia (2000): 3/5
Van Ommeren et al. (43)	Refugee camp survey	A mixed population of 810 tortured and non-tortured Bhutanese refugees in Nepal (1997)	Women: 25% Mean age: 44 y Mean duration of displacement: >5 y	Lifetime and 12-month prevalence of psychiatric disorders	CIDI (ICD-10) Administered by trained interviewers in native language	Lifetime prevalence (non- tortured refugees) PTSD: 14% PPD: 29% MDD: 8% GAD: 12% SP: 29% DD: 5%	Nepal (1997): 4/5
Kalafi et al. (44)	Structured interviews	81 Afghan refugees residing in the city of Shiraz, Iran (2002)	Women: 0% Mean age: 29y	Depression Psychiatric Problems	GHQ-28 Administered by medical student in native language	Depression: 34.6% Psychiatric Problems: 34.6%	Afghanistan (2002): PTS-A 4/5; PTS-S 5/5
Sabin et al. (10)	Cross-sectional, household survey	170 Guatemalan refugees in 5 Mayan camps in Chiapas, Mexico (2000)	Women: 58% Mean age: 38y Exposure to traumatic events: experienced 8.3 vs. observed 9.7 Mean duration of displacement: 8 years	PTSD Depression Anxiety	HTQ (Algorithm based on DSM-IV to determine cut-off points for PTSD diagnoses) HSCL-25 Administered by trained interviewers in native language	PTSD: 12% Depression: 39% Anxiety: 54%	Mexico (2000): 3/5
Lopes Cardozo et al. (45)	Stratified, systematic, random sampled survey	495 Karenni (Burmese) refugees in 3 Thai-Burmese border camps (2001)	Women: 58% Age: 15–34 y (61% of participants) Mean duration of displacement: 6–10 y (49% of participants)	PTSD Depression Anxiety	Constructed questionnaire including an adapted, event-specific version of the HTQ, 16 questions for PTSD symptoms according to the DSM-IV HSCL-25 Administered by trained interviewers in native language	PTSD: 5% Depression: 41% Anxiety: 42%	Myanmar (2001): 4/5
Kamau et al. (46)	Refugee camp survey	A mixed population of 1,852 African refugees in Kakuma, Northwest Kenya (1997–1999)	Sudanese 60% Somali 25% Ethiopian 15% Other demographics N/A	Incidence of CMD	Diagnosis was based on DSM-IV Interviews conducted by trained mental health workers in native language	PTSD: 39% Anxiety: 23% Psychosis: 12% Depression: 11% Others: 9%	Kenya (1999): 4/5
Karunakara et al. (47)	Cross-sectional survey	3,323 Ugandan nationals, Sudanese nationals, and Sudanese refugees in northern Uganda and southern Sudan (1999–2000)	Women: 75% Mean age: 30 y >1 episode of forced displacement (76% of participants)	PTSD	PCL-C (DSM-IV) Administered by trained interviewers in native language	*PTSD* Sudanese nationals: 48% Sudanese refugees: 46% Ugandan nationals: 18%	Uganda (2000): 4/5 Sudan (2000): 5/5
Thapa et al. (48)	Cross-sectional, household survey	290 Nepalese internally displaced people in Nepal (2003)	Women: 39% Mean age: 41 y Exposure to traumatic events: 4.6 Mean duration of displacement: 28.6 months	PTSD Depression Anxiety	Modified HTQ; PCL-C HSCL-25 Generalized anxiety, depression and PTSD modules of CIDI in order to validate the HSCL-25 and PCL-C Administered by trained interviewers in native language CIDI modules administered by a trained physician	PTSD: 53% Depression: 80% Anxiety: 81%	Nepal (2003): 4/5
Kim et al. (49)	IDPs camp survey	1,274 internally displaced adult females in 6 registered IDPs camps in Nyala District, Darfur Province (2005)	Mean age: 34 y Mean duration of displacement: 6 months	Depression Suicidal ideations Suicide attempts (over the previous year)	PHQ-9 Administered by trained interviewers in native language	Depression: 31% Suicidal ideation: 5% Attempted suicide: 2% Committed suicide: 2%	Sudan (2005): 5/5
Vinck et al. (50)	Multistage, stratified, random cluster survey	2,585 internally displaced people in 4 districts (villages and camps) of northern Uganda (2005)	Women: 50% Mean age: 37 y 61% of participants live in camps Exposure to traumatic events: 3.8	PTSD Depression	PCL-C HSCL-25 Administered by trained interviewers in native language	PTSD: 74% Depression: 44%	Uganda (2005): 5/5
Roberts et al. (51)	Multistage, stratified, random cluster survey	1,210 internally displaced people in 2 districts of northern Uganda (2006)	Women: 60% Mean age: 35 y Exposure to traumatic events: 8 (49% of women, 71% of men) Duration of displacement: 5–10 y (40% of participants)	PTSD Depression	Culturally adapted HTQ HSCL-25 Administered by trained interviewers in native language	PTSD: 54 % Depression: 67%	Uganda (2006): 4/5
Onyut et al. (52)	Cross-sectional survey	1,425 Somali and Rwandese Refugee in Nakivale, southwestern Uganda (2003)	Mean age: 32 y Exposure to traumatic events: 9 (Rwandese refugees) vs. 12 (Somali refugees) Mean duration spent in camps: >4 y	PTSD Depression Anxiety	PDS HSCL-25 Appropriate CIDI modules used in order to validate the PDS and HSCL-25 Administered by trained interviewers in native language CIDI administered by a clinician	*PTSD* Somali refugees: 48% Rwandese refugees: 32% Depression: N/A Anxiety: N/A	Uganda (2003): 4/5
Roberts et al. (11)	Cross-sectional, random cluster survey	1,242 refugees and internally displaced people in Juba, southern Sudan (2007)	Women: 51% Mean age: 33 y Status: 13% previously displaced as refugees; 10% previously displaced as IDP; 12% displaced more than once Exposure to traumatic events: ≥1 (92% of participants) vs. ≥8 (23% of participants)	PTSD Depression	HTQ HSCL-25 Administered by trained interviewers in native language	PTSD: 36% Depression: 50%	Sudan (2007): 5/5
Hamid and Musa (53)	IDPs camps survey	430 internally displaced persons in north and south Darfur (2005)	Women: 49% Mean age: 34 y Duration of displacement: >1 y (83% of participants)	PTSD General distress	PCL-C Administered by trained interviewers in native language	PTSD: 54%	Sudan (2005): 5/5
Husain et al. (54)	Cross-sectional multistage cluster sample survey	1,409 internally displaced people in Jaffna District, Sri Lanka (2009)	Women: 62% Mean age: 40 y Exposure to traumatic events: 2.6	PTSD Depression Anxiety	HTQ HSCL-25 Administered by trained interviewers in native language	PTSD: 7% Depression: 22% Anxiety: 33%	Sri Lanka (2009): 5/5
Richards et al. (55)	Cross sectional survey	109 internally displaced people in Medellin, Colombia (2008)	Women: 60% Mean age: 38 y Duration of displacement: 1 y (22% of participants)	PTSD Depression Anxiety	Culturally validated 24-items PTSD Checklist Zung Depression Scale Zung Anxiety Scale Self-administered	PTSD: 88% Depression: 41% Anxiety: 59%	Colombia (2008): 5/5
Roberts et al. (56)	Cross-sectional multistage cluster sample survey	1,206 internally displaced people in 2 northern Uganda districts (2006)	Women: 60% Mean age: 35y Exposure to traumatic events: ≥8 (58% of participants) Mean duration of displacement: >5y (70% of participants)	General Alcohol disorders	AUDIT Questionnaire Administered by trained interviewers in native language	Alcohol disorders: 32% (Men), 7% (Women)	Uganda (2006): 4/5
Akinyemi et al. (57)	Cross-sectional survey	Mixed population of 971 refugees and non-refugees (46% of refugees; 57% of non-refugees) in the only UNHCR camp in South-western Nigeria (2012)	Women: 40% Mean age: 35 y Mean duration of displacement: 8.6 y	Common mental disorders (CMD)	MINI Administered by trained interviewers in native language	*Refugee populations*: Depression: 45% PTSD: 34% Obsession: 34% Mania: 26% Auditory hallucinations: 21% Visual hallucinations: 13% Alcohol abuse: 13% Drug abuse: 20% Suicidal ideation: 11%	Nigeria (2012): 4/5
Salah et al. (58)	Cross-sectional survey	1,876 internally displaced people in 2 settlements in Central Sudan (2010)	Women: 56% Median age: 35 y Mean duration of displacement: 18 y	CMD	MINI (DSM-IV, ICD-10) Administered by trained interviewers in native language	MDE: 24% GAD: 23% Dysthymia: 20% Social phobia: 14% PTSD: 12% Agoraphobia: 6% PD: 5% OCD: 5% HE: 3% Alc. Dep./SD: 2%/1% Psychosis: 1.0% APD: 1% Suicidality: 0.5%	Sudan (2010): 5/5
Doocy et al. (59)	Cross-sectional survey	8681 Iraqi refugees in Jordan and Syria (2008-2009)	Women: 52% Mean age: 56 y Mean duration of displacement: 2 y 87% of participants displaced by conflict in Iraq	Depressive symptoms	HSCL-25 Administered by Iraqi and Jordanian interviewers in native language	*Overall mental disability* Jordan: 2% (81% conflict related) Syria: 5% (95% conflict related) *Depression* (% cases of mental disability) Jordan: 72% Syria: 75%	Jordan (2008): 4/5 Syria (2008): 4/5
Siriwardhana et al. (60)	Cross-sectional survey	450 internally displaced people in Sri Lanka (2011)	Women: 63% Mean age: 47 y	CMD PTSD	PRIME-MD PHQ CIDI Administered by trained interviewers in native language	CMD: 19% SD: 14% DS: 7% MDD: 5% PTSD: 3% AD: 1% Drug use: 1%	Sri Lanka (2011): 5/5
Makhashvili et al. (61)^a, b^ Roberts et al. (62) Comellas et al. (63)	Cross-sectional survey	A mixed population of 3600 IDPs and former IDPs in Georgia (2011)	Women: 65% Mean age: 38 y Exposure to traumatic events: >3 (34%)	PTSD Depression Anxiety Alcohol disorders	TSQ PHQ-9 GAD-7 AUDIT Administered by trained interviewers in native language	PTSD: 23% Depression: 13% Anxiety: 11% Hazardous alcohol use: 7% Episodic heavy drinking: 3%	Georgia (2011): 3/5
Elhabiby et al. (64)	Cross-sectional survey	74 internally displaced people in the Nyala Province, South Darfur	Women: 89% Mean age: 33 y Exposure to traumatic events: NA Mean duration of displacement: NA	Axis-I mental disorders	SCIDI-I (DSM-IV-TR) Administered by psychiatrists	PTSD: 15% Depression: 11% PTSD and depression: 8% Schizophrenia: 4% Depression with psychotic features: 3% Somatization: 3% Adjustment disorders: 3% Separation anxiety: 1% Alcoholism: 1%	South Sudan (2013): 5/5
Sheikh et al. (65)	Cross-sectional survey	258 internally displaced people in Kaduna, Northwestern Nigeria (2013)	Women: 52% Mean age: 39 y Exposure to traumatic events: 11-15 (58%)	PTSD Depression	HTQ CIDI Administered by trained interviewers in native language, under the supervision of medical professionals	PTSD: 42% PTSD and depression: 27% Depression: 9%	Nigeria (2013): 4/5
Vukovic et al. (66)	3-year follow-up with clinical interviews	534 Bosnian refugees in Varazdin, Croatia (1996-1999)	Women: 59% Age: 35-54 y (34%)	PTSD Depression	HTQ HSCL-25	PTSD and depression: 21% Depression: 18% PTSD: 6%	Croatia (1996): 3/5
Llosa et al. (67)	Cross-sectional survey using a double-sampling method	326 selected refugees in Burj el-Barajneh camp, Lebanon (2010)	NA	Axis-I mental disorders	MINI Clinical reappraisals by an experienced psychologist after general screening by trained interviewers. All interviews conducted in native language	*Crude prevalence*: MDD: 31% (lifetime) Dysthymia: 4% (current) Suicidality: 12% (current) Manic Episode: 5% (lifetime) HE: 2% (lifetime) PD: 41% (lifetime) Agoraphobia: 2% (current) Social phobia: 1% (current) OCD: 4% (current) PTSD: 4% (current) Psychotic disorders: 7% (lifetime) Mood with psychotic features: 1% (current) GAD: 8% (current)	Lebanon (2010): 3/5
Alpak et al. (68)	Psychiatric interviews	352 Syrian refugees in a tent city in Gaziantep city, Turkey (2013)	Women: 49.1% Mean age: 38 y Exposure to traumatic events: 3.7 Duration of displacement: 6.52 m	PTSD	PTSD (DSM-IV-TR)—Psychiatric interview Administered by trained psychiatry resident in native language	PTSD: 45.2% lifetime prevalence, 33.5% current prevalence	Syria (2013): 5/5
Feyera et al. (69)	Cross-sectional survey	847 adult Somali refugees in Melkadida, Southeast Ethiopia (2014)	Women: 54% Median age: 33 y Exposure to traumatic events: 4-7 (41%)	Depression Substance abuse	PHQ-9 Semi-structured questionnaire Administered by trained interviewers in native language	Depression: 38% Current substance use (i.e., last 3 months): Alc. 3%, Khat 31%, Cigarette 23%	Ethiopia (2014): 4/5
Naja et al. (70)	Cross-sectional survey	310 Syrian refugees registered with selected NGOs in Beirut and Mount Lebanon, Lebanon (2014)	Women: 61.2% Age: 25-44 (66% of participants)	Depression	MINI Administered by trained interviewers in native language	Depression: 43.9%	Syria (2014): 5/5
Kazour et al. (71)	Household survey	452 Syrian refugees in 6 refugee camps in Central Bekaa region, Lebanon (2017)	Women: 55.8% Mean age: 35 y Duration of displacement: 10 m	PTSD Substance abuse disorders	MINI MINI Administered by mental health professionals in native language	PTSD: 35.4% lifetime prevalence, 27.2% current prevalence Substance abuse disorders: 1.99% lifetime prevalence, 0.66% current prevalence	Syria (2016): 5/5

a Roberts et al. (62), Makhashvili et al. (61), and Comellas et al. (63) use the same subjects from a cross-sectional survey

b Makhashvili et al. (62) used results from 3,025 subjects who completed the cross-sectional survey.

*Alc. Dep./SD, Alcohol Dependence/Substance Dependence; AD, Anxiety Disorders; Audit, Alcohol Use Disorder Identification Test; APD, Antisocial Personality Disorder; CMD, Common Mental Disorders; CIDI, Composite International Diagnostic Interview; DD, Dissociative Disorder; DS, Depressive Syndromes; DSM, Diagnostic and Statistical Manual of Mental Health Disorders; GAD-7, Generalized Anxiety Disorder instrument; HE, Hypomanic Episode; HSCL-25, Hopkins Symptoms Checklist 25; HTQ, Harvard Trauma Questionnaire; IDP(s), Internally Displaced People/Persons; MINI, Mini-International Neuropsychiatric Interview; MDD, Major Depressive Disorder; MDE, Major Depression Episode; N/A, Non Available; OCD, Obsessive Compulsive Disorder; PCL-C, PTSD Checklist–Civilian Version; PD, Panic Disorder; PDS, Post-traumatic Diagnostic Scale; PHQ-9, Patient Health Questionnaire; PPD, Persistent Pain Disorder; PRIME-MD PHQ, Primary Care Evaluation of Mental Disorders Patient Health Questionnaire; PTS, Political Terror Scale; PTSD, Post-Traumatic Stress Disorder; SCIDI-I, Structured Clinical Interview for DSM-IV-TR axis I disorders; SD, Somatoform Disorders; SP, Specific Phobia; SRQ, Self Reporting Questionnaire; TSQ, Trauma Screening Questionnaire; UNHCR, United Nations High Commissioner for Refugees*.

### Origin of studies

Conflict-affected regions and host countries of displaced populations were unevenly represented: six original studies were conducted in the Middle East (Iran, Syria, Jordan, Lebanon, and Turkey) (44, 59, 67, 68, 70, 71), two were conducted in Latin America (Mexico, Colombia) (10, 55), six in Central and Eastern Europe (Croatia and Georgia) (38, 40, 61–63, 66), eight in Asia (Thailand, Myanmar, Sri Lanka, Nepal, China, and Cambodia) (36, 37, 41, 43, 45, 48, 54, 60), and 16 in Africa (Sudan, Uganda, Liberia, Nigeria, Kenya, The Gambia, Senegal, and Ethiopia) (11, 39, 42, 46, 47, 49–53, 56–58, 64, 65, 69). The majority of studies were conducted in countries with a PTS score of 4 (*n* = 13) or 5 (*n* = 18).

### Demographics

With regard to demographic factors, 26 studies (10, 11, 36, 38–41, 45, 47, 50, 51, 54–56, 58–66, 69–71) reported on samples that consisted of a majority (50–75%) of internally displaced or refugee women. Mean age ranged between 30 and 40 years in 21 studies (10, 11, 36, 38, 39, 42, 43, 46, 47, 49–52, 58, 60, 63–65, 68, 69, 71). In terms of status of displacement, our systematic research yielded quasi-similar results: IDPs and refugees were the primary population of interest in 16 countries (11, 48–51, 53–56, 58, 60–65) and 16 studies (10, 11, 36, 38, 40, 42, 43, 45–47, 52, 57, 59, 66, 67, 69), respectively. The mean duration of displacement ranged from 6 months to 18 years (10, 36, 38, 42, 43, 45, 47–59). Although the strict UNHCR concept of *protracted situation* was not used as an inclusion criterion, nine studies selected in this review described durations of displacement >5 years (10, 36, 43–45, 51, 56–58). Levels of traumatic exposure among forcibly displaced populations were significant and reached an average number of seven experienced and/or witnessed traumatic events per participant (10, 11, 37, 38, 40, 42, 48, 50–52, 54, 56, 61, 65, 69). These events included different forms of psychological stressors: witnessing the murder of a family member, friend, or stranger; being close to death, tortured, beaten, or arbitrarily imprisoned; being violently separated from family or isolated from other social groups, raped or sexually abused, and forced to accept ideas against one's will (11, 46).

### Assessment methods

Most eligible studies included in our review used well-established mental health questionnaires administered by trained lay interviewers or medical practitioners. Questionnaires were systematically assessed for cultural validity, translated, and administered in the participants' native language. The HTQ (33) was the preferred assessment method and was used in almost 50% of the studies looking into the prevalence of PTSD [15 of 33 studies; (10, 11, 36–42, 48, 51, 54, 65, 66)]. Anxiety disorders and depression were mostly assessed through the HSCL-25 [17 of 31 studies; (34)] (10, 11, 36–39, 41, 42, 45, 48, 50–52, 54, 59, 66). With regard to PTSD, depression, and anxiety disorders, several authors also reported the Composite International Diagnostic Interview [CIDI; (72)] as a cross-culturally validated assessment method consisting of structured clinical interviews based on DSM-IV and ICD-10 diagnostic criteria (43, 48, 52, 60, 65). In two distinct studies, clinicians administered specific CIDI modules in order to validate the diagnoses mentioned above (48, 52). Common mental disorders were assessed either by means of the Mini-International Neuropsychiatric Interview (MINI) (57, 58, 67, 70, 71), a structured clinical diagnostic interview according to the DSM-IV and ICD-10, or the CIDI (43, 60). One group of experts based their evaluation of axis-I mental disorders on the Structured Clinical Interview for DSM [SCID-I; (64)]; administered by trained psychiatrists. Definitive clinical diagnoses were based on validated diagnostic methods such as DSM-III-R, DSM-IV, DSM-IV-TR, and ICD-10 (40, 46, 68). Seven studies measured alcohol related problems and reported five different screening methods: the CAGE questionnaire (58), the AUDIT questionnaire (40)—a more detailed set of questions developed by the World Health Organization (WHO) to detect hazardous drinking—MINI standard interviews (56, 62), structured psychiatric interviews focused on alcohol abuse based on the DSM-III or DSM-IV (50, 57, 71), and the SCID-I (40).

### Prevalence of psychiatric disorders: PTSD, depression, and anxiety disorders

PTSD, depression, and anxiety disorders were the most common psychiatric illnesses investigated. However, prevalence rates of PTSD were disproportionately represented in 30 studies and consistently high: data varied between 2.2 and 88.3% (64). We found depression and anxiety disorders to be the second and third most commonly reported mental disorders among IDPs and refugees: specific estimates varied from 5.1 to 81% for depression (10, 11, 36–43, 45–48, 50–55, 57, 58, 60–65, 68, 71) and from 1 to 90% for anxiety disorders (10, 11, 36–39, 41, 42, 44–46, 48–52, 54, 55, 57–67, 69, 70). Estimates of more precise diagnoses of anxiety disorders were compatible with the high prevalence of unspecified anxiety among displaced populations. One study showed that generalized anxiety disorder, social phobia, and obsessive-compulsive disorder affected 23, 14, and 5%, respectively, of a sample of 1,876 long-term IDPs in Central Sudan (58).

### Prevalence of psychiatric disorders: common mental disorders

Fifteen studies looked beyond the general concepts of trauma, PTSD, anxiety, and depression in settings of conflict-related forced displacement (40, 43, 44, 46, 49, 56–64, 67, 69, 71). These studies considered additional conditions such as substance abuse, psychosis, suicidality, personality disorders, and other forms of mood and anxiety disorders.

Alcohol use disorders were the most common type of substance abuse reported and were particularly prevalent among displaced men (2–60%) (40, 56–58, 62, 64). A Croatian study described rates of non-comorbid alcohol dependence as high as 60.5% (40). Conversely, although drug abuse reached 20% in one recent survey assessing common mental disorders (CMD) in a mixed population of IDPs and refugees in South-western Nigeria (57), drug abuse generally did not exceed 2% (58, 60, 71).

Psychotic disorders were explored in two different samples of African IDPs and refugees and one selected group of refugees in Lebanon: data were heterogeneous and prevalence ranged between 1 and 12% (46, 58, 67). Psychotic symptoms such as visual or auditory hallucinations, however, presented in one African study were as high as 13 and 21%, respectively (57).

We identified four studies completed in Sudan, Southwestern Nigeria, and Lebanon that investigated suicidality, representing a population of 4,447 adult IDPs and refugees (49, 57, 58, 67). In one recent study conducted in a refugee camp in Lebanon by the French NGO *Médecins sans Frontières*, current rates of suicidality reached 12% (67). Similar results were observed in a Nigerian refugee camp (57). A survey examining the health status of internally displaced adult females in Darfur reported a prevalence rate of 2% for more specific suicidal behaviors, namely attempted or committed suicide (49).

Prevalence of pain disorders was 29% in a group of 392 non-tortured Bhutanese refugees in Nepal (43). Similarly, somatoform disorders were examined in only one survey, conducted in Sri Lanka, and found to affect 14% of 450 IDPs (60).

## Discussion

In recent years, there has been a growing interest in research activities related to the psychiatric health sequela of armed conflicts. The high number of people affected globally by organized violence and the low level of available knowledge justify the growth in both quality and quantity of these activities. This systematic review examined for the first time the prevalence of common and uncommon psychiatric disorders among IDPs and refugees displaced as a consequence of armed conflicts in LMICs. The results suggest that PTSD, depression and anxiety disorder are highly prevalent after displacement and armed conflicts. This association can be partially accounted for by distinct psychosocial vulnerabilities of IDP and refugee populations.

This review highlights a lack of studies assessing the prevalence of mental health disorders among forcibly displaced populations in conflict-affected middle–eastern countries as only six studies originated from these regions. This result is particularly striking in view of the ever-changing and ever-increasing figures of worldwide forced migration. For example, according to a 2017 *UNHCR* report (60), countries such as Turkey, Pakistan, Lebanon, or the Islamic Republic of Iran hosted more than 28% of the world's refugees, people who had been affected from the ongoing conflicts in the Syrian Arab Republic or Afghanistan.

The detailed analysis of the studies included in this review showed a high variability in the duration of displacements between studies. However, we observed that the UNHCR definition of *protracted situation* was never used as a strict methodological consideration. Rates of trauma exposure were found to be not only high in terms of prevalence but also in terms of recurrence and intensity: all participants included in the reviewed studies had experienced or witnessed at least one serious traumatic event. Thirty-one studies were conducted in populations displaced from countries with a PTS score of four or five. Although prevalence of disorders reported across these countries were heterogeneous, the point estimates for those displaced from countries with a PTS score of less than three were on average lower than those in the highest two PTS quintiles. Understanding how unstable political situations with forced displacement relate to heightened rates of CMDs could inform the development of targeted interventions. Women tended to be over-represented in the studies included in this review, irrespective of their displacement status. This is in line with other literature in refugee and IDP populations (73). The traumas faced after displacement differ between men and women, and the effects of these traumas may manifest different (74). Additionally, this may have caused variance in the reported prevalence's of common psychiatric disorders, which have been shown to differ between men and women (e.g., substance-abuse disorders and depression) (75).

Our findings confirm a long-standing inclination of mental health research toward PTSD, depression, and anxiety disorders in settings of complex emergencies. Public mental health research conducted over the past 20 years has largely focused on the immediate psychological aftermaths of armed conflicts in light of the well-described associations between these psychiatric disorders, displacement, and generalized forms of violence. Demographic and socio-economic characteristics of displaced populations are known to be potent moderators of mental health: migration, especially internal displacement, protracted conflict situations, and economic instability are strongly associated with poor mental health outcomes (5).

We point out a *substantial* lack of data concerning the general mental health conditions of forcibly displaced populations in LMICs, which might be caused by different mechanisms (28): (1) stigma of mental disorders in developing countries, (2) disproportionate under-representation of several conflict-affected regions in the literature—such as Latin America, Central and Eastern Africa, Central Asia—(3) cultural or political barriers to assessment *and* implementation of mental health programs and policies such as insufficient funding of mental health research, (4) over-centralization of mental health resources, (5) severe shortage of adequately trained mental health staff, and, finally, (6) weak public health leadership in the field of mental health. The very few estimates about substance abuse, other mood and anxiety disorders, psychosis, or suicidality are higher than figures from the general population studies conducted in LMICs (13, 14, 76–84).

Lastly, there is a relative consensus on how to assess common mental disorders among IDPs and refugees. Structured questionnaires such as the HSCL-25, HTQ, PCL, and CIDI are largely favored for the evaluation of PTSD, depression, and anxiety disorders; they are also systematically examined for cultural validity, translated into the participants' native language, and administered by trained lay people, mental health specialists or medical practitioners. Conversely, other forms of mental disturbances such as psychosis or suicidality are assessed through MINI or SCID interviews with the help of the DSM or ICD for diagnosis validation.

## Limitations

This review has a number of limitations and should therefore be interpreted cautiously. Despite our systematic search strategy, it is likely that certain unpublished or non-indexed studies have not been included. We did not establish direct contacts with authors in order to identify additional studies. Furthermore, the generalizability of our results to the entire IDP or refugee population is limited. Most research groups have favored the cross-sectional study that has been repeatedly criticized for its limitations in terms of selection bias and causality analysis (85–87). The proportion of psychiatric cases pre-existing the conflict and directly caused or at least influenced by armed conflicts and subsequent displacements remains unclear due to a lack of mental health programs in LMICs.

The presented results were extracted from studies conducted in several regions that differ greatly in terms of population characteristics, culture, social traditions, religion, language, and even types of conflict or duration of displacement. No meta-analysis has been undertaken, therefore, inter-study variability and publication bias have not been examined, and heterogeneity has not been significantly reduced. Moreover, the cross-cultural aspect of a majority of studies may have influenced the final results (88).

The HSCL-25 and HTQ have been extensively used to assess depression, anxiety disorders, and PTSD among IDP sub-populations and refugees, but not validated for general populations in LMICs (89). Many validation studies have been conducted in novel contexts for these scales and others, but it is imperative that cultural adaptations be completed prior to their use in novel contexts (33, 90). In addition, both the HSCL-25 and the HTQ only report on symptoms. Therefore, it remains unclear to what extent these symptoms would correlate with formal clinical diagnoses based on the DSM or ICD.

Finally, some of studies did not specify whether any cultural adaptation was undertaken to formulate diagnostic criteria. Psychiatric presentation and diagnosis (including the criteria outlined in the DSM and ICD), are known to exhibit cross-cultural differences (91). Due to the recent refugee and humanitarian crises, psychometric measures and clinical interviews used to ascertain the presence of CMDs (i.e., depression, anxiety, and PTSD) in refugee populations have been widely studied and validated (92). In relation to the severe and uncommon disorders reported in this study, there is a paucity of research in the refugee context. As such, it is possible that authors used culturally unadapted methods to determine diagnosis and that estimated prevalence rates may not be representative of the true values in the populations studied.

## Practical implications and conclusion

Wars and other forms of organized violence generally draw the attention of policymakers, mass media, and non-governmental organizations. Mental health and public health experts tend to consider this selective and often temporary attention as an opportunity to raise awareness about the psychological consequences of armed conflicts, namely PTSD, to warn about the disastrous mental health situation in low- and middle-income countries. With the numbers of displaced individuals reaching unprecedented levels, a more global mental health approach is necessary to effectively support affected nations. In places where violence is seen as a necessary factor in achieving peace, ongoing armed conflict, and displacement will likely contribute to continued psychological impairment and suffering among those affected (50). Allowing for a better understanding of the effects that the aftermath of war have on the psychological well-being will allow for interventions not only targeting mental illness but also attitudes toward reconciliation and justice and reduction of future violence (93).

In sum, this systematic review indicates that the heterogeneity in prevalence rates is caused by methodological differences between studies and differences between conflict-affected IDP and refugee populations. We recommend that future public mental health research goes beyond the assessment of PTSD, depression, and anxiety disorders and consider a broader inclusive definition of the psychological consequences of armed conflict as additional key concept. In addition to that questionnaires assessing more severe disorders (e.g., psychotic disorders) which are often ignored need to be developed and validated for use in LMICs.

Ambitious and locally coordinated assessment programs of mental health should be implemented as well as non-centralized mental health policies and their systematic qualitative evaluations (94). Lastly, the on-going crises indicate that there is an urgent need for scalable interventions that are appropriate for war-torn contexts in which resources are limited. The World Health Organization is currently spearheading this with the so called low-intensity intervention Problem Management Plus [PM+; (95)] being the most evidence-based release to date (96, 97). On-going research is aiming to increase the scope of this intervention to allow for group-based and application-based administration. However, the most critical challenge will be to translate these promising intervention programs into sustainable public mental health policies in countries so deeply weakened by protracted conflicts, destruction of fragile pre-existing health care structures, and political instability. Finally, it is unfortunately common that refugees and IDPs are not being treated fairly wherever they end up seeking protection and support, and are subjected to ongoing humiliating, traumatizing, or otherwise damaging circumstances. From a societal and ethical viewpoint, changing these circumstances may even constitute a higher priority than diagnosing and treating trauma-related mental illness.

## Author contributions

All authors contributed to the study conception and design. AA and NM conducted literature search and data extraction. NM and AA performed data analyses and drafted the manuscript. All authors contributed to and approved the final version of the manuscript.

### Conflict of interest statement

The authors declare that the research was conducted in the absence of any commercial or financial relationships that could be construed as a potential conflict of interest.
